# Prognostic value of lymph node ratio in stage III non-small-cell lung cancer: A retrospective cohort study

**DOI:** 10.1097/MD.0000000000035341

**Published:** 2023-10-06

**Authors:** Xiang Zhang, Nan Feng, Bo Wu, Yiping Wei, Wenxiong Zhang

**Affiliations:** a Department of Thoracic Surgery, The Second Affiliated Hospital of Nanchang University, Nanchang, China.

**Keywords:** lymph node ratio, node-positive, non-small cell lung cancer, survival, TNM

## Abstract

A growing number of studies have found that the lymph node ratio (LNR) is an important indicator of prognosis in non-small-cell lung cancer (NSCLC). Impact analysis for LNR was performed for survival in patients undergoing surgery for stage III NSCLC compared to the surveillance, epidemiology and end results databank. Clinicopathological variables, such as cancer-specific survival (CSS), were taken from the surveillance epidemiology and end result databank of stage III NSCLC patients who underwent surgery, and the LNR threshold stratification of NSCLC patients was computed by X-tile. CSS was assessed by the Kaplan–Meier method with CSS-independent risk factors calculated by multivariate Cox regression analysis. In total, 7011 lung cancer patients were included. Multifactorial analysis showed that LNR and positive node category had predictive value for stage III NSCLC. In patients with stage IIIA NSCLC, Kaplan–Meier analysis demonstrated that patients with T1-2N2 stage had clearly superior CSS than those with T3-4N1 stage (*P* < .001), which conflicted with the results from the assessment of primary tumor, lymph nodes, and metastasis/N stage. The cutoff values for LNR were 0.31 and 0.59. Kaplan–Meier analysis demonstrated that the CSS was substantially better in patients with LNR-low than in those with LNR-medium or LNR-high (*P* < .001), which was also proven by multivariate competing risk regression. Subgroup analysis suggested that the survival advantage of a lower LNR was achieved in all subgroups (sex, race, etc). In stage III NSCLC, the LNR is a valuable factor for assessing prognosis, in which a higher LNR indicates a worse prognosis.

## 1. Introduction

Lung cancer currently ranks second in the world in terms of occurrence, comprising 11.4% of cancers worldwide with 21.3% of deaths from cancer worldwide, and lung cancer leads the world in new cases and deaths.^[1]^ Non-small cell lung cancer (NSCLC) accounts for about 80% of all lung cancers, and about 20% to 35% of NSCLC cases are diagnosed as stage III. However, the main tool for predicting patient prognosis after surgery in stage III patients is N staging, and there are different N stagings for stage III due to different primary tumor, lymph node, and metastasis (TNM) stages, and patients with different N stagings have different prognosis. Predicting the prognosis of patients by the number of involved lymph nodes is not an ideal solution. Therefore, in order to find a tool that can accurately predict the prognosis prediction of patients undergoing surgery for stage III NSCLC, we opted for the lymph node ratio (LNR), this categorizes patients into low-risk, medium-risk, and high-risk groups based on its varying values. By doing so, this approach seeks to enhance prognostic accuracy, offering valuable insights for clinical decision-making. LNR is considered a better prognostic stratification tool and has been shown to predict salivary gland cancer, gastric cancer and oral prognosis of patients with squamous cell carcinoma.^[2–4]^ Although there are many studies demonstrating the prospective significance of LNR in NSCLC patients, the size of these studies is not sufficient to derive an optimal LNR cut point.^[5,6]^

To research whether the LNR predicts the outcome of patients with stage III NSCLC during this study, we used cancer-specific survival (CSS) and other pathological variables from the surveillance, epidemiology and end results (SEER) databank for patients treated surgically for stage III NSCLC as well as computed cutoff values for LNR using the X-tile method. The cutoff scores of the optimal LNR were used to further stratify lymph node-positive NSCLC patient profiles.

## 2. Methods

### 2.1. Data sources and study population

The SEER project, managed by the National Cancer Institute, is one of the largest public databases that collect cancer incidence data from their population-based cancer registries, and based on the 2010 census, this publicly available SEER database covers nearly 27.8% of the United States. We extracted 7011 NSCLC patients who died of cancer from 2004 until 2018 from the SEER databank and searched for data on the following variables: age, tumor primary site, race, positive node category (pN) classification, histologic subtype, grade, sex, CSS and survival months, regional nodes examined, regional nodes positive, and positive tumor. NSCLC patients were reclassified in accordance with the 8th edition TNM classification. Criteria for admission included the table below: Patients were NSCLC patients; Patients with TNM stage III NSCLC; Every patient received a lung cancer surgical resection; All patients underwent intraoperative lymph node dissection. Criteria for elimination included the following: Pathological classification as small cell lung cancer; None of the patients underwent surgery; Intraoperative lymph node dissection was not performed; and Radiotherapy or chemotherapy treatment was performed. Patient statistics according to the American Joint Committee on cancer classification were examined and graded according to the 8th version.^[7]^

### 2.2. Statistical analysis

Data statistics were divided into the following steps. In step one, the LNR cutoff figure was calculated with X-tile^[8]^ and then stratified into 3 subgroups of low, medium and high LNR. In step 2, survival rates were computed for the different subgroups with corresponding *P* values along with hazard ratios (HR) with the help of the Kaplan–Meier approach.^[9]^ In step 3, 2 Cox regression models are constructed including LNR and pN respectively as well as multivariate regression analysis, making the corresponding forest plots. Cox regression models were able to assess variables with significant effects on CSS in multivariate analysis, and HR with 95% confidence intervals (CI) were calculated, where *P* < .05 in the Cox regression represents a significant difference in statistical terms.^[10]^ In step 4, akaike information criteria (AIC) values of Cox regression types, including LNR, were compared with those of Cox regression types, including pN, and the smaller AIC values yielded a better suitability of the model and allowed the best model to be derived.^[11]^ In describing the data of interest, the absolute number, mean, standard deviation (SD), 25th percentile (Q1), 75th percentile (Q3), and median of the data were calculated. The above data analysis was conducted with the software R version 4.1.2.^[12]^

### 2.3. Patient and public involvement

None.

## 3. Results

### 3.1. Clinicopathologic characteristics

**Figure 1.**
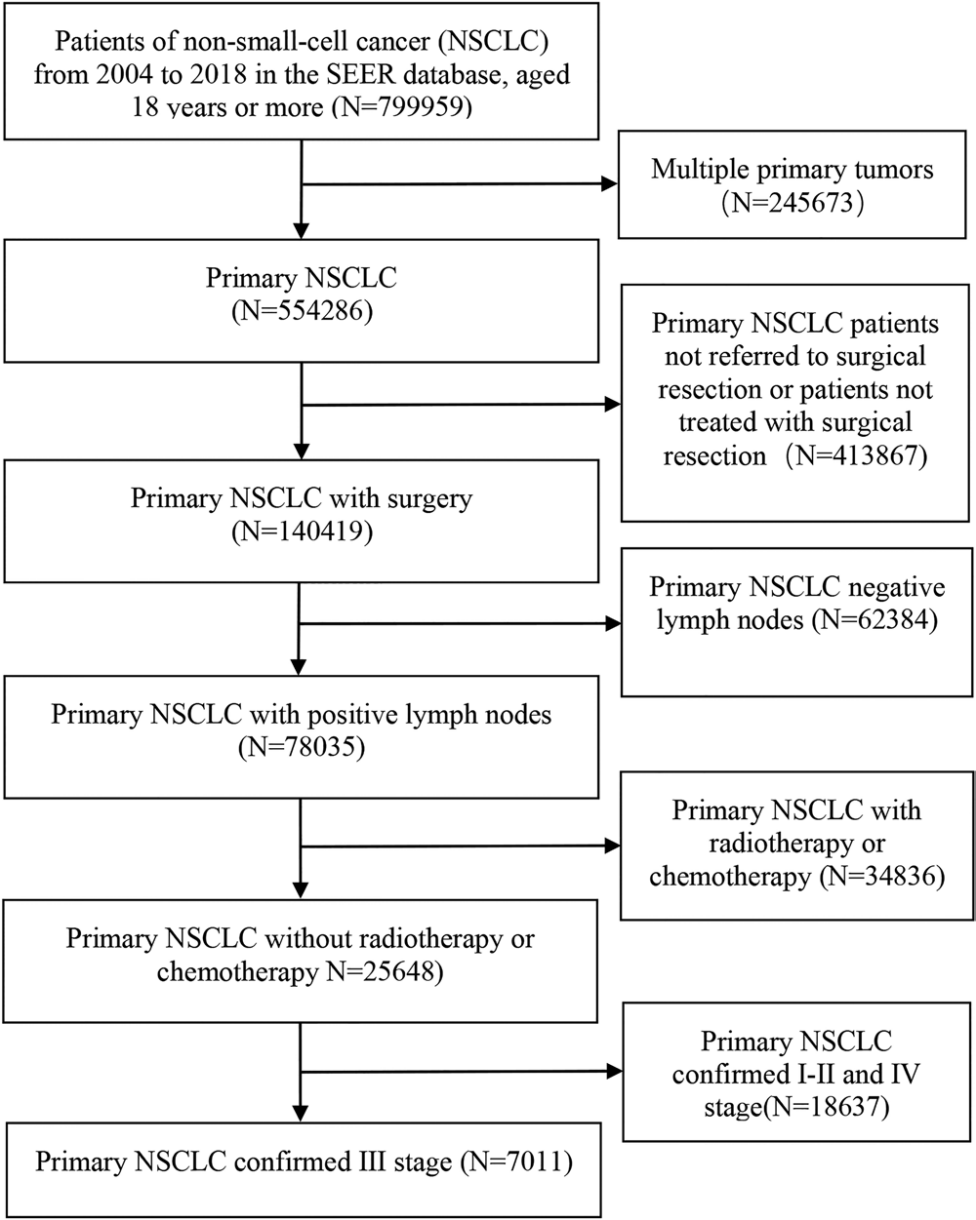
Research flow chart.

The process of study flow chart can be seen in Figure 1. We extracted 614355 patients diagnosed with NSCLC from the SEER databank from January 1, 2004 until December 31, 2018. The ultimate research group was composed of 7011 patients following the application of exclusion and acceptance factors. The study cohort is shown in Table 1 for both demographic and benchmark features. Throughout the study, it included a range of histological isoforms, such as adenocarcinoma (N = 4100, 58.5%), squamous cell carcinoma (N = 1715, 24.5%) and other NSCLC isoforms (N = 1196, 17%). Patients aged 60 years or older comprised the majority of patients with NSCLC (66.0%); in addition, N1, N2, and N3 accounted for 18.5%, 80.1% and 1.4% of patients, respectively. The CSS patients had a median duration of 75 months of follow-up (95% CI: 73.0, 77.0).

**Table 1:** Baseline characteristics of patients with stage III non-small cell lung cancer with positive lymph nodes.

Characteristics	No. (%)
Sex	
Female	3434 (49.0)
Male	3577 (51.0)
Race	
White	5707 (81.4)
Black	664 (9.5)
Others	640 (9.1)
Age	
≤60	2386 (34.0)
>60	4625 (66.0)
Primary site	
Upper lobe	4043 (57.7)
Lower lobe	2260 (32.2)
Main bronchus	140 (2.0)
Middle lobe	308 (4.4)
Overlapping/NOS	260 (3.7)
Histology	
Adenocarcinoma	4100 (58.5)
Squamous	1715 (24.5)
Others	1196 (17)
T stage	
T1	1482 (21.1)
T2	3085 (44.0)
T3	896 (12.8)
T4	1548 (22.1)
N stage	
N1	1296 (18.5)
N2	5613 (80.1)
N3	129 (1.4)

N = lymph node, No. = number, NOS = no obvious syndrome, T = tumor.

**Figure 2.**
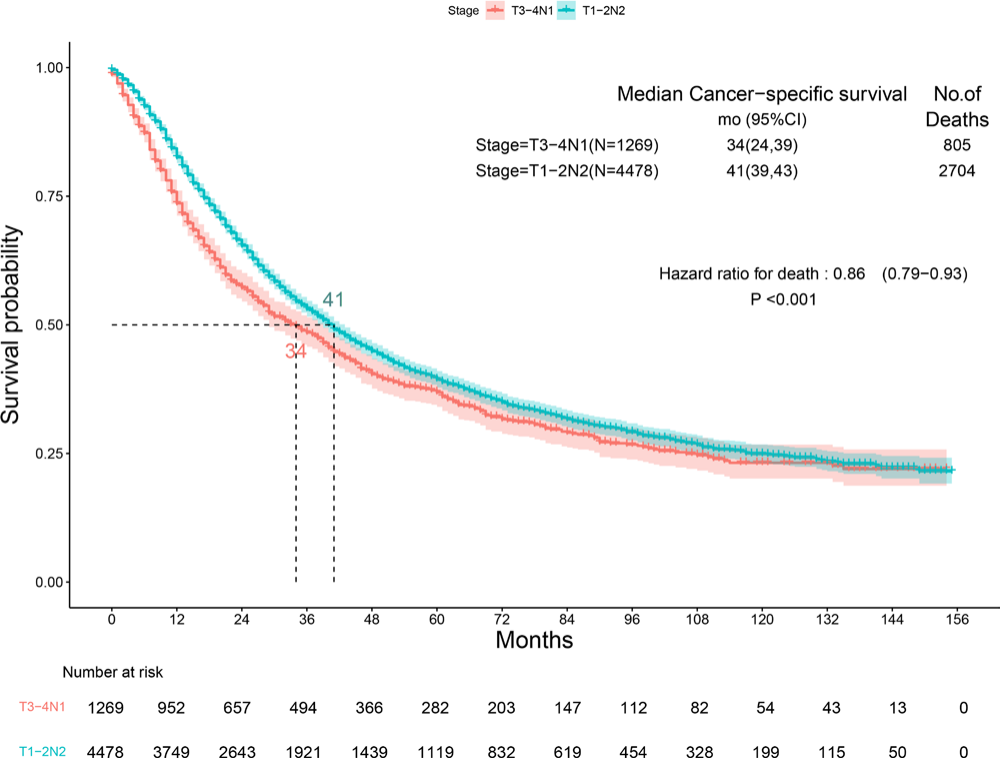
Kaplan–Meier survival curves for stage T3-4N1 patients versus stage T1-2N2 patients.

In total, 4402 patients died of NSCLC over the follow-up period. The median CSS of the included cases was 36 months (range 0–155). The median CSS ranged from 39 months for IIIA (range 0–155), 27 months for IIIB (range 0–155) and 11 months for IIIC (range 0–155) (Supplemental Fig. 1, http://links.lww.com/MD/K39). Because of TNM staging, stage IIIA accounted for the largest proportion, and N staging included N0, N1, and N2. We compared patients undergoing surgery with N staging lymph node positivity, of whom 1269 were T3-4N1 patients and 4478 were T1-2N2 patients. Among stage IIIA NSCLC cases, the Kaplan–Meier plot showed that for IIIA patients treated surgically, stage N2 had a longer mean postoperative survival time than stage N1 (Fig. 2), although both belong to stage IIIA, T staging may have a greater impact on patients.

### 3.2. Analysis of LNR cutoff points and comparison with pN stage

**Figure 3.**
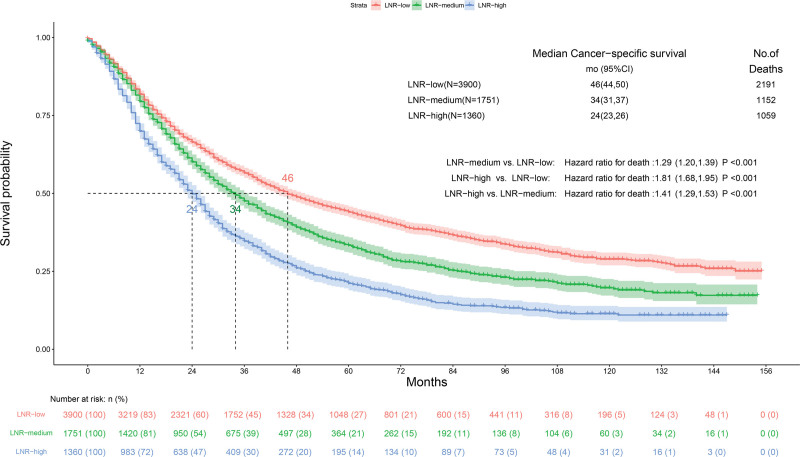
The best cutoff values for the lymph node ratio (LNR) by using X-tile.

**Figure 4.**
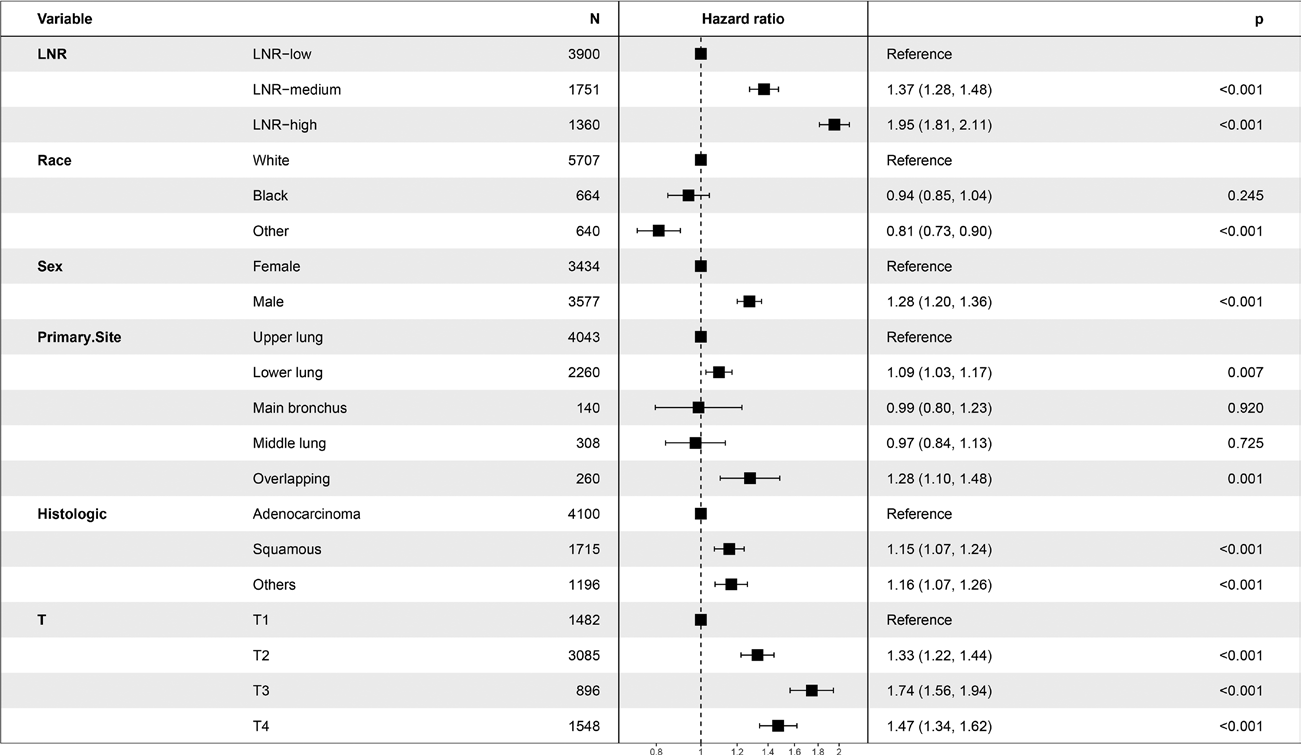
Forest plot demonstrating the results of multivariate Cox regression analysis of cancer-specific survival (CSS) predicted by lymph node ratio (LNR) as a covariate.

**Figure 5.**
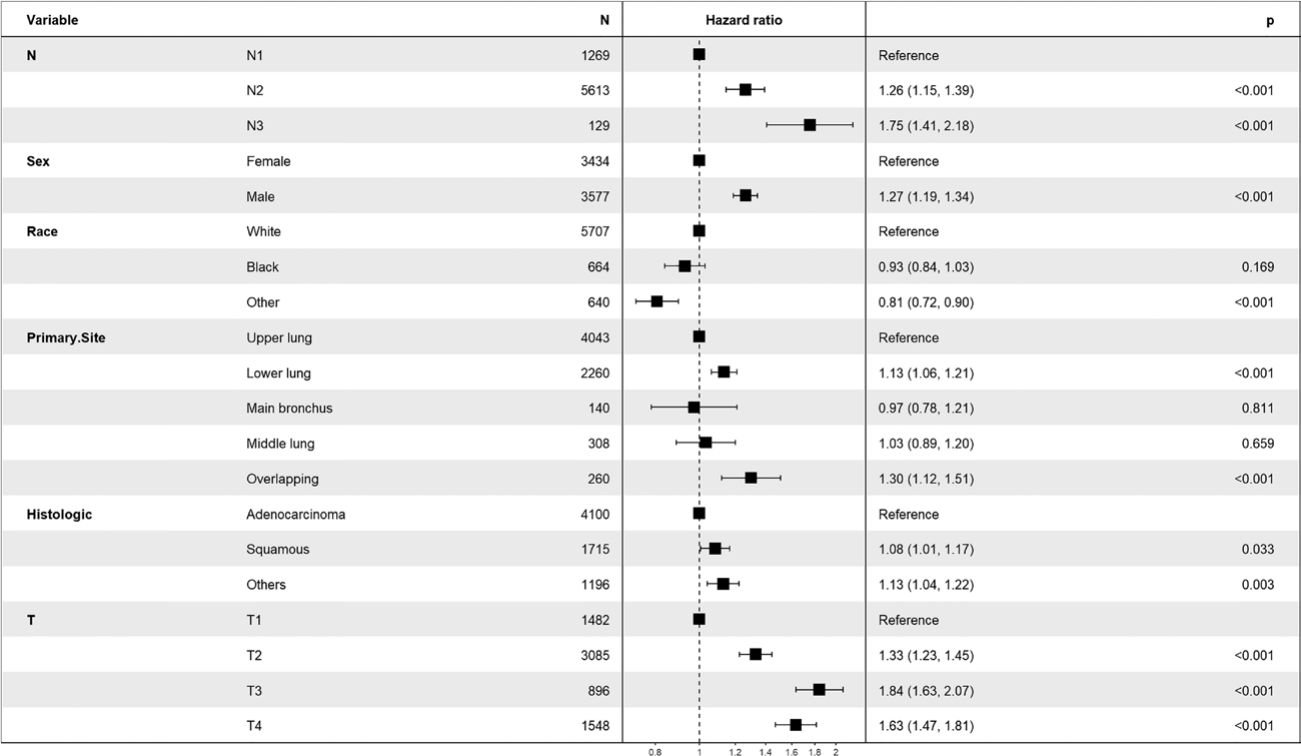
Forest plot demonstrating the results of multivariate Cox regression analysis of cancer-specific survival (CSS) predicted by positive node category (pN) as a covariate.

The median retrieved regional nodes examined was 10 (Q1. Q3: 6.17) and the median positive regional nodes retrieved was 2 (Q1. Q3: 1.4). The median LNR was 0.28 (Q1. Q3: 0.14. 0.50). The CSS database of the SEER databank of NSCLC patients with CSS data was analyzed by X-tile and demonstrated low (LNR-low ≤ 0. 31; n = 3900, 55.6%), medium (0.31 < LNR-medium ≤ 0.59; n = 1751, 25.0%), and high (LNR-high > 0.59; n = 1360, 19.4%). Kaplan–Meier analysis to predict CSS in patients with low LNR and in patients with medium and high LNR (Fig. 3). We further demonstrated that a higher LNR as a covariate acted an independent as well as a significant adverse correlate of CSS by applying multivariate Cox regression to LNR (LNR-high vs LNR-low: HR: 1.95, 95% CI: 1.81–2.11, *P* < .001) (Fig. 4). We selected pN to be the covariate using multivariate Cox regression to demonstrate that higher pN is likewise a separate as well as a clearly poor predictor for CSS (N3 vs N1: HR: 1.75, 95% CI: 1.41–2.18; *P* < .001) (Fig. 5).

Analysis of a multivariate competitive risk regression model constructed with LNR together with pN demonstrates that pN also represents an independent as well as important predictor of CSS (Table 2). The LNR can also be an essential factor in forecasting CSS on its own (Table 2) (LNR-high vs LNR-low: sub-distribution HR: 1.94, 95% CI: 1.80–2.10, *P* < .001; LNR-medium vs LNR-low: sub-distribution HR: 1.35, 95% CI: 1.25–1.45, *P* < .001). The AIC suggested that the predicted effects of LNR appeared to be better than those of pN (8902.229 vs 9133.555).

**Table 2:** Competing risk regression analysis included lymph node ratio (LNR) and pN stage separately as covariates.

Characteristics	LNR group		pN group	
	SHR	*P*	SHR	*P*
LNR classification				
LNR-low	1.00 (Reference)		—	—
LNR-medium	1.35 (1.25, 1.45)	<.001	—	—
LNR-high	1.94 (1.80, 2.10)	<.001	—	—
N stage				
N1	—	—	1.00 (Reference)	
N2	—	—	1.24 (1.12, 1.43)	<.001
N3	—	—	1.77 (0.98, 2.12)	<.001
Sex				
Female	1.00 (Reference)		1.00 (Reference)	
Male	1.27 (1.20, 1.35)	<.001	1.25 (1.18, 1.33)	<.001
Race				
White	1.00 (Reference)		1.00 (Reference)	
Black	0.93 (0.84, 1.03)	.178	0.92 (0.83, 1.02)	.118
Others	0.78 (0.71, 0.87)	<.001	0.78 (0.70, 0.87)	<.001
Primary site				
Upper lobe	1.00 (Reference)		1.00 (Reference)	
Lower lobe	1.05 (0.98, 1.12)	.172	1.08 (1.01, 1.16)	.018
Main bronchus	0.86 (0.68, 1.08)	.188	0.85 (0.68, 1.07)	.159
Middle lobe	0.95 (0.82, 1.10)	.477	1.04 (0.87, 1.17)	.881
Overlapping/NOS	1.26 (1.07, 1.49)	.005	1.30 (1.10, 1.53)	.002
Histology				
Adenocarcinoma	1.00 (Reference)		1.00 (Reference)	
Squamous	1.16 (1.08, 1.26)	<.001	1.09 (1.01, 1.18)	.023
Others	1.18 (1.09, 1.28)	<.001	1.15 (1.06, 1.25)	.001
T stage				
T1	1.00 (Reference)		1.00 (Reference)	
T2	1.32 (1.22, 1.43)	<.001	1.32 (1.22, 1.43)	<.001
T3	1.69 (1.51, 1.89)	<.001	1.75 (1.54, 1.98)	<.001
T4	1.44 (1.31, 1.58)	<.001	1.58 (1.42, 1.75)	<.001

LNR = lymph node ratio, N = lymph node, NOS = no obvious syndrome, pN = positive node category, SHR = sub-distribution hazards ratio, T = tumor.

### 3.3. Prognostic stratification of pN disease according to LNR conclusion

Subgroup analysis showed that a lower LNR had a survival advantage in all subgroups (sex, race, N stage, etc) (Table 3). Therefore, we would like to further attempt to clarify the association that exists between the LNR with pN stage. The reason for this is the involvement of lymph nodes is a major determinant of clinical outcome and determines the therapy options for patients with surgically treatable NSCLC. We analyzed data on CSS in the N1 group of NSCLC patients using X-tile and found the N1 subgroup to have an excellent LNR cutoff of 0.11 as well as 0.32. The N1 subgroups were low (LNR-low ≤ 0.11; n = 370, 29.2%), medium (0.11 < LNR-medium ≤ 0.32; n = 574, 45.2%), and high (LNR-high > 0.32; n = 325, 25.6%) in the 3 subgroups (Supplemental Fig. 2A, http://links.lww.com/MD/K41). According to the continuous distribution of LNR, a higher LNR is related to a higher RR (Supplemental Fig. 2B–C, http://links.lww.com/MD/K41). Analysis of CSS in the N2 group of NSCLC patients by X-tile analysis found that the N2 subgroup had excellent LNR cutoffs of 0.24 and 0.57. These 2 cutoffs classified the N2 subgroup as low (LNR-low ≤ 0.24; n = 240, 39.9%), medium (0.24 < LNR-mid ≤ 0.57; n = 2151, 38.3%), and high (LNR- high > 0.57; n = 1222, 21.8%) in the 3 subgroups (Supplemental Fig. 3A–C, http://links.lww.com/MD/K45). Analysis of CSS in the N3 group of NSCLC patients by X-tile analysis found that the N3 subgroup had an excellent LNR cutoff of 0.27 and 0.64. These 2 cutoffs classified the N3 subgroup into low (LNR-low ≤ 0.27; n = 38, 29.5%), medium (0.27 < LNR-mid ≤ 0.64; n = 39, 30.2%), and high (LNR-high > 0.64; n = 52, 40.3%) subgroups (Supplemental Fig. 4A–C, http://links.lww.com/MD/K47), and Kaplan–Meier analysis demonstrated that in all 3 groups, the CSS was considerably higher in patients with a low LNR than in those with an LNR-medium or LNR-high (*P* < .001) group (Supplemental Fig. 5A–C, http://links.lww.com/MD/K49). We also tried to subgroup analysis the patients with the same LNR value, and found that the postoperative survival time of patients with stage N1 was greater than that of patients with stage N2 at the same LNR value of 0.5 by Kaplan–Meier analysis (Supplemental Fig. 6, http://links.lww.com/MD/K51).

**Table 3:** The univariate multi-subgroup Cox regression model of LNR.

Variable	Number	Low cutoff	High cutoff	LNR-low	LNR-medium		LNR-high	
		Points	Points	HR (95% CI)	HR (95% CI)	*P*	HR (95% CI)	*P*
Total	7011	0.31	0.59	1.00 (Reference)	1.37 (1.28, 1.48)	<.001	1.95 (1.81, 2.11)	<.001
Race								
White	5707	0.26	0.57	1.00 (Reference)	1.26 (1.17, 1.36)	<.001	1.82 (1.67, 1.97)	<.001
Black	664	0.09	0.58	1.00 (Reference)	1.49 (1.02, 2.16)	.038	2.36 (1.57, 3.54)	<.001
Other	640	0.18	0.71	1.00 (Reference)	1.60 (1.25, 2.04)	<.001	2.38 (1.71, 3.31)	<.001
Sex								
Female	3434	0.35	0.64	1.00 (Reference)	1.42 (1.28, 1.59)	<.001	1.96 (1.75, 2.18)	<.001
Male	3577	0.15	0.59	1.00 (Reference)	1.31 (1.19, 1.45)	<.001	1.96 (1.74, 2.20)	<.001
Primary site								
Upper lung	4043	0.35	0.78	1.00 (Reference)	1.48 (1.35, 1.62)	<.001	1.95 (1.73, 2.20)	<.001
Lower lung	2260	0.14	0.59	1.00 (Reference)	1.22 (1.06, 1.41)	<.001	1.97 (1.68, 2.31)	<.001
Main bronchus	140	0.32	0.54	1.00 (Reference)	2.01 (1.22, 3.01)	.006	1.30 (0.66, 2.55)	.449
Middle lung	308	0.24	0.58	1.00 (Reference)	0.75 (0.52, 1.07)	.11	1.66 (1.18, 2.34)	.004
Overlapping	260	0.11	0.44	1.00 (Reference)	1.60 (1.00, 2.56)	.049	2.35 (1.43, 3.86)	<.001
Histological type								
Adenocarcinoma	4100	0.35	0.75	1.00 (Reference)	1.44 (1.31, 1.57)	<.001	2.12 (1.90, 2.37)	<.001
Squamous	1715	0.1	0.33	1.00 (Reference)	1.24 (1.05, 1.46)	.0119	1.82 (1.54, 2.16)	<.001
Others	1196	0.26	0.53	1.00 (Reference)	1.29 (1.09, 1.53)	.003	1.76 (1.48, 2.10)	<.001
T stage								
T1	1482	0.32	0.59	1.00 (Reference)	1.41 (1.19, 1.66)	<.001	2.28 (1.92, 2.70)	<.001
T2	3085	0.16	0.55	1.00 (Reference)	1.38 (1.23, 1.55)	<.001	2.04 (1.79, 2.32)	<.001
T3	896	0.09	0.54	1.00 (Reference)	1.35 (1.08, 1.69)	.008	2.55 (1.91, 3.40)	<.001
T4	1548	0.26	0.56	1.00 (Reference)	1.45 (1.27, 1.66)	<.001	1.95 (1.63, 2.33)	<.001
N stage								
N1	1269	0.11	0.32	1.00 (Reference)	1.00 (0.84, 1.19)	.973	1.56 (1.30, 1.87)	<.001
N2	5613	0.24	0.57	1.00 (Reference)	1.30 (1.20, 1.40)	<.001	1.91 (1.75, 2.08)	<.001
N3	129	0.27	0.64	1.00 (Reference)	1.55 (0.89, 2.69)	.125	2.30 (1.37, 3.83)	<.001
TNM stage								
IIIA	5747	0.24	0.59	1.00 (Reference)	1.29 (1.20, 1.39)	<.001	1.87 (1.71,2.04)	<.001
IIIB	1224	0.32	0.76	1.00 (Reference)	1.35 (1.16, 1.57)	<.001	1.80 (1.50,2.15)	<.001
IIIC	40	0.42	0.93	1.00 (Reference)	2.35 (0.99, 5.57)	.053	1.68 (0.73,3.86)	.224

CI = confidence interval, HR = hazards ratio, LNR = lymph node ratio, N = lymph node, NOS = no obvious syndrome, T = tumor, TNM = primary tumor, lymph nodes, and metastasis.

## 4. Discussion

We carried out an analysis based on the SEER database of 7011 NSCLC patients based on the population. The findings revealed that the higher the LNR was, the worse the patient outcome. We also found that N2 staging showed a superior prognosis compared to N1 staging for patients undergoing surgery at stage IIIA. Therefore, the most important conclusion we obtained was that the LNR represents an important separate predictive element in lymph node-positive patients. We can use this value to help stratify patient risk. And in stage IIIA, T staging could exert a more significant impact on patient prognosis than N staging. Consequently, N staging might not accurately predict patient prognosis in certain stages.

In clinical practice, TNM staging is often used for cancer staging in NSCLC patients to select the appropriate treatment strategy, but studies at this stage have found significant differences in postoperative survival in stage IIIA patients, although they are all graded as stage IIIA. For stage IIIA patients, the size of the tumor probably matters more than lymph node metastasis, especially in the N2 group.^[13–15]^ Currently, our classification of pN has been performed upon the metastasis in the lymph nodes attached to the primary tumor, which gradually spread to far away lymph nodes, for example, axillary lymph nodes, but at this stage, it has been shown that some patients who skipped lymph node metastasis at the beginning of stage N2 had a longer survival time than those who metastasized from stage N1 to stage N2. Our study also found that even for NSCLC patients with the same stage IIIA, their prognosis was also better for patients with N2 stage than for patients with N1 stage.^[16–20]^ The N stage cannot accurately predict patient prognosis for NSCLC patients in the same TNM stage, while LNR is more accurate than pN in predicting patient prognosis, and the larger the tumor in IIIA is, the more influential it may be on patient outcome.^[21]^

N staging is an important factor in the survival of patients with NSCLC, and lymph node count and site count are 2 proxies of the N staging.^[22]^ Both indicators result primarily from surgical harvesting of the lung as well as the mediastinal lymph nodes and the pathology of lymph nodes and sample analysis by pathologists after the procedure. Therefore, there are some technical issues regarding the use of these 2 indicators for prognostic stratification. Surgical treatment of premature NSCLC should consist of removal of a minimum of 10 lymph nodes. However, lymph nodes are sometimes not removed intact, and accurate prognostic stratification is not possible due to limitations in the extent of surgery and limitations in detection techniques, as well as variations caused by the different strengths of different hospitals and testing facilities.^[23]^ Therefore, LNR indices are increasingly important in the prognostic indices of NSCLC patients. Research suggests the LNR is an isolated predictive element of CSS, which can stratify the prognosis of patients with different N stages and help patients choose a more appropriate treatment. We found several trials that support the usefulness of the LNR in NSCLC patient outcomes. According to Qiu C. et al, the LNR has been identified for surgically excised stage I-III small cell lung cancer as an independent predictive factor, suggesting that a higher LNR correlates with lower survival.^[9]^ The prognostic value of the LNR should be verified further in future studies. According to Taylor M.D. et al, increased LNRs in patients undergoing NSCLC excision were independently associated with decreased survival and shorter time to return following R0 excision.^[10]^ This was concluded to correlate the survival of patients in early-stage NSCLC to the number of removed LNs. To provide a more accurate clinical staging judgment, we believe that the LNR is more appropriate than the pN classification for the selection of clinical treatment strategies. We believe that the LNR can minimize influencing factors and avoid interference from external factors.^[24]^

Compared to previous studies, our study has some strengths and limitations. These studies cover postoperative treatment with adjuvant treatment (radiotherapy or chemotherapy),^[5]^ which may affect the postoperative survival time of patients, indicating that the data obtained from their studies do not truly express the normal evolution of NSCLC and the actual survival time after surgery, whereas our study excluded patients with postoperative adjuvant therapy at the beginning of the screening, which makes our survival data and results independent of other factors. In addition, the X-tile method of determining the best cutoff was used, which will make the cutoff more scientific and precise. In addition, we compared the N staging of patients undergoing this stage IIIA surgery and found that the larger the tumor, the worse the patient prognosis is likely to be at the same stage.^[6]^ There are a few drawbacks to this study. This study is based on SEER database data and requires external validation of the LNR threshold. Incomplete adjuvant therapy data hinder the use of LNR for predicting postoperative survival in all NSCLC patients. We are establishing our hospital database to explore LNR values in lymph node-positive NSCLC patients for future research, aiming to verify LNR predictive ability in clinical practice and improve postoperative survival and treatment plans for patients.

## 5. Conclusion

In summary, LNR serves as a crucial predictive indicator for patients with stage III NSCLC who undergo surgical treatment without receiving chemotherapy or radiotherapy. In stage IIIA NSCLC, N2 patients have better prognosis than N1 patients, suggesting larger tumors have a stronger impact on survival, and N staging might not predict survival accurately in some stages. Stratification for LNR by X-tile allows for more accurate prediction of postoperative survival time for surgical patients than N staging and allows for more effective selection of appropriate clinical treatment options.

## Acknowledgments

The authors would like to thank all public health workers of cancer statistics, all patients and doctors relevant to this research.

## Author contributions

**Conceptualization:** Xiang Zhang, Nan Feng, Bo Wu, Wenxiong Zhang.

**Data curation:** Xiang Zhang, Nan Feng, Bo Wu.

**Formal analysis:** Xiang Zhang, Nan Feng, Bo Wu, Wenxiong Zhang.

**Funding acquisition:** Wenxiong Zhang.

**Investigation:** Xiang Zhang, Nan Feng, Bo Wu.

**Methodology:** Xiang Zhang, Nan Feng, Bo Wu, Yiping Wei.

**Project administration:** Xiang Zhang.

**Resources:** Xiang Zhang.

**Software:** Xiang Zhang.

**Validation:** Yiping Wei, Wenxiong Zhang.

**Visualization:** Yiping Wei, Wenxiong Zhang.

**Writing – original draft:** Xiang Zhang, Wenxiong Zhang.

**Writing – review & editing:** Xiang Zhang, Yiping Wei, Wenxiong Zhang.

## Supplementary Material
