# Supplementary figures and images for: Prognostic value of lymph node ratio in stage III non-small-cell lung cancer: A retrospective cohort study
Source: Medicine (Baltimore). 2023 Oct 6;102(40):e35341. doi: 10.1097/MD.0000000000035341 (PMC10553147; doi:10.1097/MD.0000000000035341)

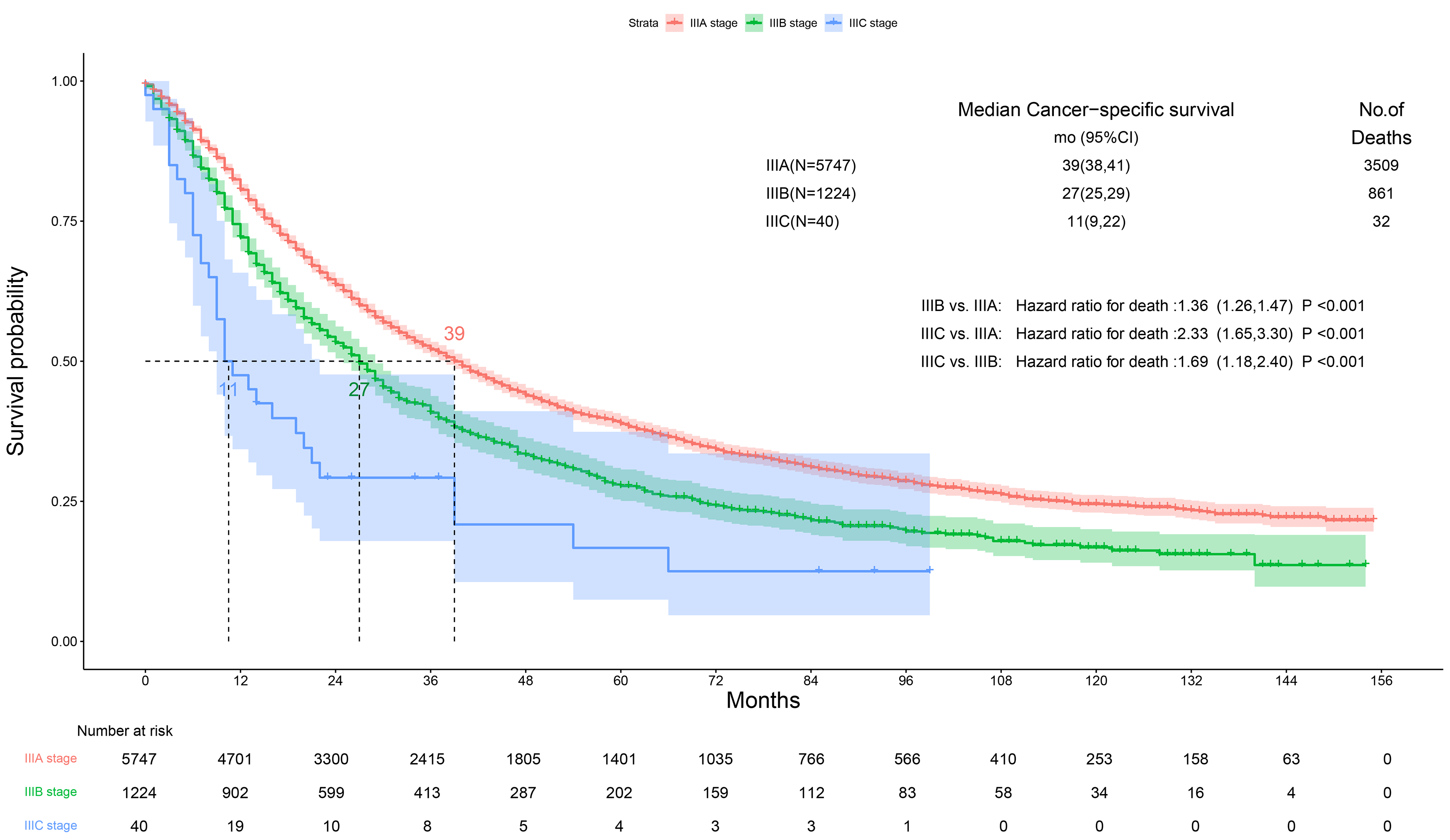

Supplement: Supplementary file 1 [file medi-102-e35341-s001.tif]

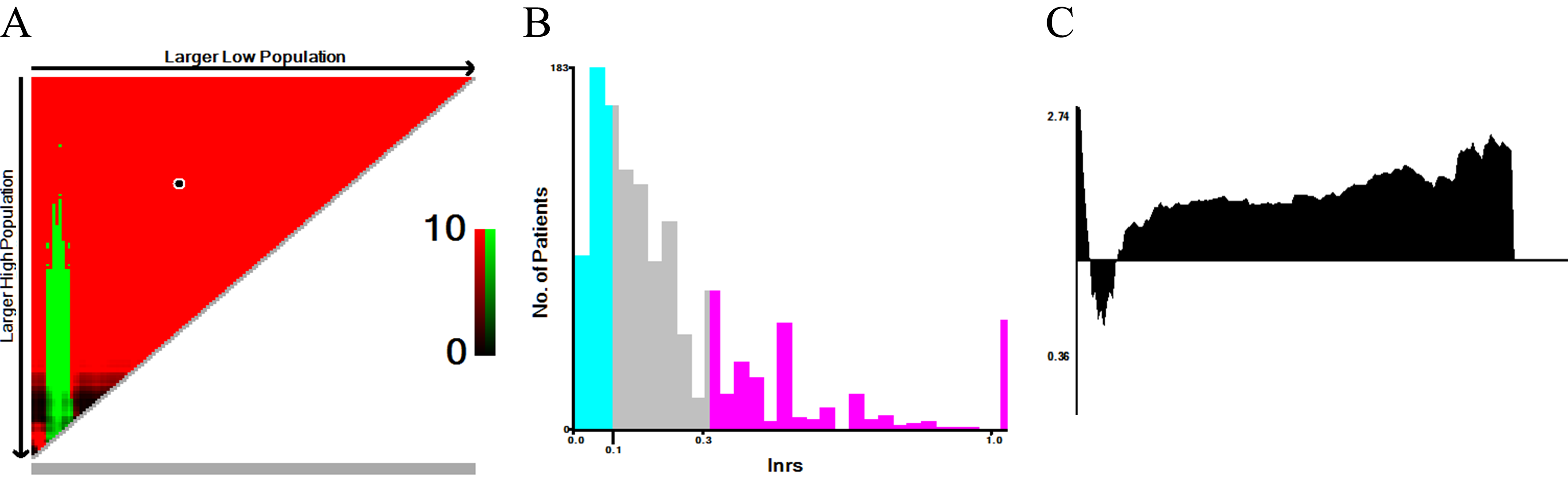

Supplement: Supplementary file 2 [file medi-102-e35341-s002.tif]

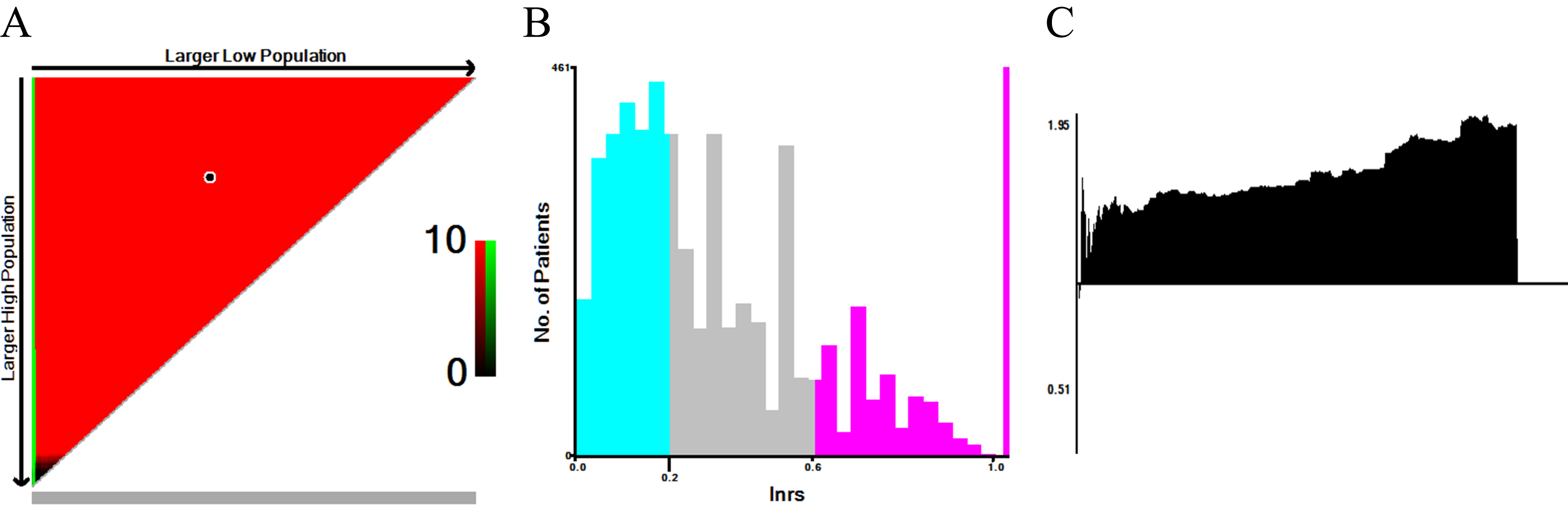

Supplement: Supplementary file 3 [file medi-102-e35341-s003.tif]

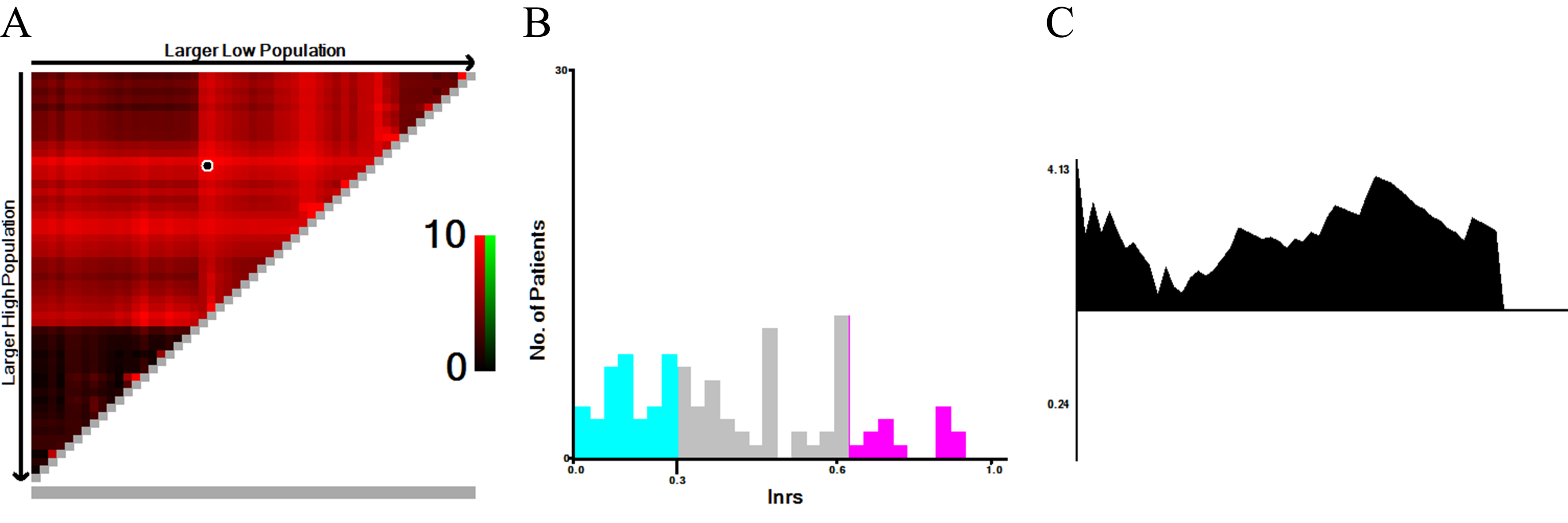

Supplement: Supplementary file 4 [file medi-102-e35341-s004.tif]

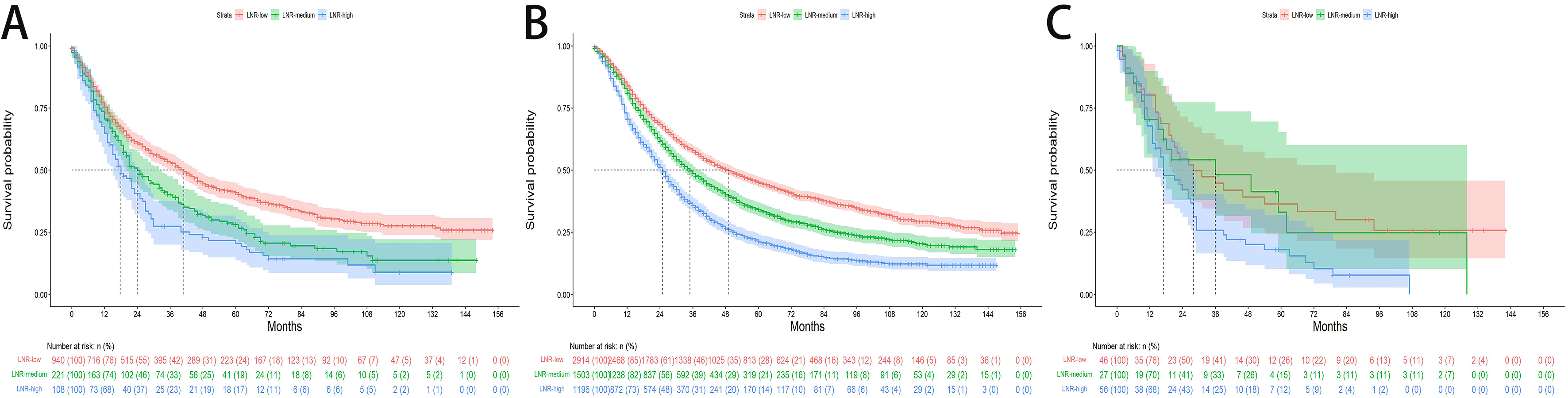

Supplement: Supplementary file 5 [file medi-102-e35341-s005.tif]

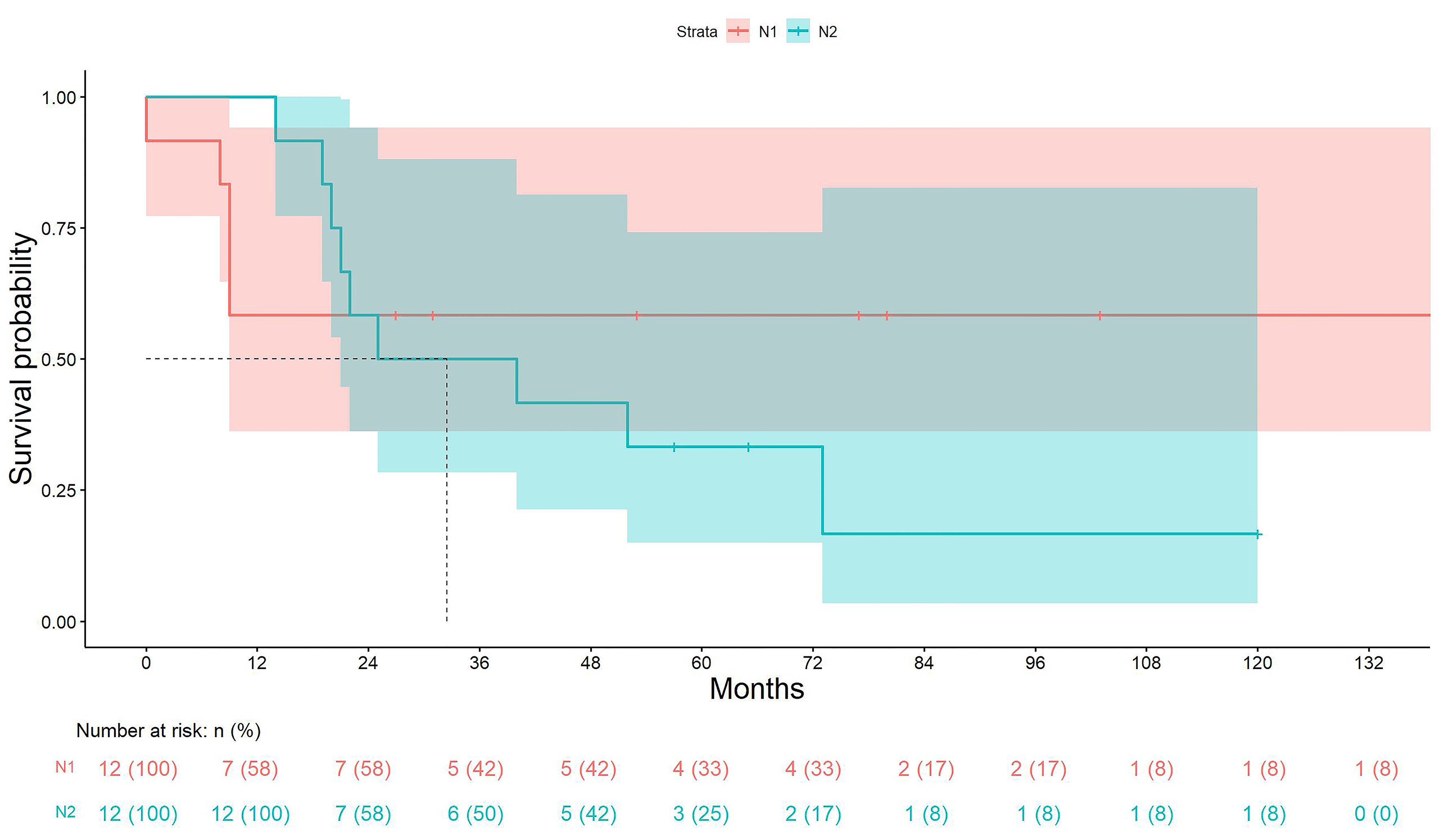

Supplement: Supplementary file 6 [file medi-102-e35341-s006.tif]
